# Impact of Long-Term Supplementation with Probiotics on Gut Microbiota and Growth Performance in Post-Weaned Piglets

**DOI:** 10.3390/ani14111652

**Published:** 2024-05-31

**Authors:** Soo-Yeon Park, Yo-Han Kim, Sung-Jae Kim, Jeong-Hee Han

**Affiliations:** 1 Department of Veterinary Pathology, College of Veterinary Medicine and Institute of Veterinary Science, Kangwon National University, Chuncheon 24341, Republic of Korea; vetsoo@ctcbio.com; 2 Department of Large Animal Internal Medicine, College of Veterinary Medicine and Institute of Veterinary Science, Kangwon National University, Chuncheon 24341, Republic of Korea; kimyohan@kangwon.ac.kr; 3 Department of Companion Animal Health, Kyungbok University, Namyangju 12051, Republic of Korea

**Keywords:** weaned piglet, probiotics, growth performance, gut microbiome

## Abstract

**Simple Summary:**

Weaned piglets must rely only on solid feed without sow milk; consequently, weaning affects the intestinal architecture and is a critical period in which the gut microbiota changes and develops in weaned piglets. This study investigated the effects of long-term probiotic supplementation (6 weeks) on the gut microbiome and growth performance in healthy weaned pigs. As a result, the growth performance of the weaned piglets with probiotics was improved compared to the piglets without probiotics. In the gut microbiome analysis, the α-diversities of the piglets with probiotics significantly increased compared to those of the piglets without probiotics. In the changes of bacterial relative abundance, the *Ruminococcaceae*, *Prevotella* and *Eubaterium coprostanoligenes* groups significantly increased in the piglets with probiotics compared to the piglets without probiotics, whereas *Muribaculaceae* significantly increased in the piglets without probiotics compared to the piglets with probiotics. Taken together, significant differences on the gut microbiome were found according to probiotic supplementation in the weaned piglets and these gut microbiome changes appeared to improve the growth performance. Thus, long-term probiotic supplementation is recommended for piglets in the nursery stage after weaning.

**Abstract:**

This study aimed to investigate effects of long-term probiotic supplementation on gut microbiota and growth performance in health weaned piglets. The non-probiotic group (N-PrB) was fed only a basal diet, while the probiotic group (PrB) was fed a basal diet + probiotic combination (*E*. *faecium* 1.6 × 10^8^ CFU/g, *B. subtilis* 2.0 × 10^8^ CFU/g, *S*. *cerevisiae* 3.0 × 10^8^ CFU/g). The probiotics combination was provided to the PrB, mixing with the basal diet in 5 kg/ton. As a result, the PrB exhibited significantly improved weight gain compared to the N-PrB (*p* = 0.00991). In the gut microbiome analysis, the PrB exhibited a significant increasing tendency of α-diversity compared to those of the N-PrB (*p* < 0.01). In the bacterial relative abundance changes in bacteria comprising the gut microbiota, *Ruminococcaceae* (*p* = 0.00281) and *Prevotella* (*p* = 0.00687) tended to significantly increase in the PrB, but decreased in the N-PrB. The *Eubaterium coprostanoligenes* group exhibited an increasing tendency in both groups, but tended to increase more significantly in the PrB compared to the N-PrB (*p* = 0.00681). *Muribaculaceae* tended to significantly increase in the N-PrB, but decreased in the PrB (*p* = 0.002779). In this study, significant differences on the gut microbiome were found according to the probiotics supplementation in the weaned piglets and these gut microbiome changes appeared to improve the growth performance.

## 1. Introduction

Probiotics have been studied extensively as feed additives in animal husbandry and have been demonstrated to benefit animal health, ultimately improving productivity [1,2]. The most frequently used bacterial genera were the lactic acid bacteria, mainly *Lactobacillus* and *Bifidobacterium*. Other genera such as *Enterococcus*, *Streptococcus*, and *Leuconostoc* are also used [3].

These probiotics benefit the host via several mechanisms. First, probiotics can modulate the host’s microbiota through the “barrier” effect to inhibit pathogenic bacterial colonization. Generally, bacteria compete each other for their habitat, inhibiting the growth of other bacteria and probiotic or commensal bacteria previously colonized in gut, which can help protect the host from colonization (infection) with more harmful microorganisms by acting as a physical barrier. This bacterial inhibition by probiotics is attributed to the production of antimicrobial substances such as bacteriocins and biosurfactants, or production of low-pH acids (not favorable for bacterial growth) induced by metabolites such as short-chain fatty acids (SCFA) [3].

Probiotics also enhance the host immune system. Over 70% of immune cells are in the gut, especially in the small intestine. Structural components repeated on the surface of microorganisms, referred to as microbial-associated molecular patterns (MAMPs), stimulate immune cells of the gut immune system at the lamina propria level [4]. In the previous study, it was revealed that probiotics increased the number of intraepithelial lymphocytes, density of CD3+ T cells in Peyer’s patches and lamina propria of jejunum, when young piglets were supplemented with probiotics for 6 weeks [5].

At 3 to 4 weeks after birth, young piglets are weaned and separated from their mothers. In this time, the piglets encounter environmental changes (place, temperature, population density, diet, etc.) in which the major factor is feed change. They must rely only on solid feed without sow milk; consequently, weaning affects the intestinal architecture (increased crypt depth, increased complexity of villus morphology, and reduced villus height) [6] and is a critical period in which the gut microbiota changes and develops in weaned piglets [7,8].

Microorganisms inhabiting the intestine are receiving considerable attention because of their fundamental functions in host health such as immune modulation, nutrient supplementation, disease prevention, and physical development [5]. Some major microbes comprise the majority of the gut microbiota and are believed to play critical roles [9]. The correlation between the gut microbiota and animal health or productivity in pigs has been well investigated in many previous studies [2,10,11,12].

Swine production cycle is generally divided into four production stages: farrowing/suckling piglet (~3–4 weeks), nursery/weaned piglet (~9–10 weeks), grower (~16 weeks) and finisher (~6 months). Upon the nursery stage, piglets are moved to a nursery facility or a wean-to-finish building with weaning event and the health of weaned piglets is likely to be poor due to several stress factors resulting from environmental changes, especially dietary change give great impact on the intestinal environment as mentioned above [7,8]. Reduced gut barrier function and increased pathogen infection, which are attributed to dysbiosis of the gut microbiota, lead to high morbidity and mortality rates. 

Taken together, the gut microbiota composed of various bacterial communities have a significant impact on the host’s internal environment affecting many functions and dysbiosis of gut microbiota could lead negative results in health and disease as mentioned above. These close relationships between gut microbiota and health have attracted great attention in probiotics use to positively modulate the gut microbiota [3]. Modulation of the gut microbiota using probiotics is considered as a promising option for sustaining a healthy gut microbiota, supported by the results of many previous studies [1,3,13]. 

Relative long-term intake of probiotics may be more effective than short-term intake, because it will take some time for probiotics to become stably established in the gut bacterial community. However, from an economic perspective of animal production industry, the longer the probiotic feeding period, the greater the cost burden on production farms. Among the swine production stages (suckling, nursery, grower and finisher), weaned piglets in the nursery stage are most likely to maintain unstable gut microbiota in their entire life. Thus, when considering both effectiveness and economic aspects, Probiotics feeding focused on this stage would be very helpful in improving the productivity of swine farms.

This study investigated the efficacy of probiotics on weaned piglets going through the nursery stage (4–10 weeks). Especially, we focused on evaluating how probiotics supplementation affects the gut microbiota and accordingly improves growth rates. Weaned piglets were supplemented with probiotics throughout the entire period of the nursery stage and, growth performance evaluation and gut microbiome analysis were carried out.

## 2. Methods

### 2.1. Ethics Statement

The use of animals and all experimental protocols (protocol number: KW-230807-2) were approved by the Institutional Animal Care and Use Committee of Kangwon National University (Chuncheon-si, Republic of Korea).

### 2.2. Experimental Design

Eighteen healthy weaned piglets (Duroc × [Landrace × Yorkshire], castrated male), which were 4 weeks old, were allocated into two groups (probiotics and non-probiotics). Water and food were provided ad libitum. All piglets were fed commercial feed at the nursery stage (basal diet) before transitioning to the grower stage, and no antibiotic therapy was administered to any piglet during the nursery stage (4–10 weeks). The non-probiotic group (N-PrB) was fed only a basal diet, while the probiotic group (PrB) was fed a basal diet + probiotic combination. The probiotics combination (*Enterococcus* (*E*) *faecium* EF-1 1.6 × 10^8^ CFU/g, *Bacillus* (*B*) *subtilis* BS-CTC 2.0 × 10^8^ CFU/g, *Saccharomyces* (*S*) *cerevisiae* SC-5 3.0 × 10^8^ CFU/g) was mixed with the basal diet in 5 kg/ton. Clinical symptoms (fever, cough, and diarrhea) were observed daily during the experimental period. To evaluate the growth performance, the body weight (BWs) of the piglets was measured at 4 and 10 weeks. For gut microbiome analysis, fecal samples were collected using a DNA/RNA shield collection tube with swabs (Zymo Research, Orange, CA, USA) at 3 and 10 weeks. All swab samples were stored at −80 °C before use.

### 2.3. 16S rRNA Sequencing

Genomic DNA was extracted from fecal samples using the QIAamp DNA Mini Kit (Qiagen, Hilden, Germany). Subsequently, approximately 469 bp encompassing the V3 and V4 hypervariable region within 16S rRNA gene were amplified with two universal primers with adapter overhang sequences: V3-F, 5-TCGTCGGCAGCGTCAGATGTGTATAAGAGAC AGCCTACGGGNGGCWGCAG-3, and V4-R, 5-GTCTCGTGGGCTCGGAGATGTGTAT AAGAGACAGGACTACHVGG GTATCTAATCC-3. 16S rRNA Sequencing was performed on an Illumina MiSeq platform (San Diego, CA, USA). Raw Illumina MiSeq data were classified using an index sequence, and a paired-end FASTQ file was created for each sample.

### 2.4. Sequence Data Processing

The sequence data were processed using QIIME2 (v. 2022.2). After adaptor removal using Cutadapt (v. 3.4), demultiplexed reads were processed and merged using the DADA2 plugin. For the paired-end reads, the forward sequence (Read 1) and reverse sequence (Read 2) were trimmed to 280 and 210 bp, respectively, and sequences with expected errors of ≥2 were then eliminated. Subsequently, an error model for each batch was established for denoising. The paired-end sequences were aligned into a single sequence, and the chimeric sequence was eliminated using the DADA2 consensus method to extract amplicon sequence variants (ASVs). Subsequently, taxonomy assignment was conducted with the SILVA silva138 AB V3–V4 classifier using the feature-classifier classify-sklearn plugin. PhlyoSeq data were generated for further analysis using R Studio (v. 4.3.1).

### 2.5. Microbial Diversity Analysis

The α-diversity refers to microbial diversity within a single sample and is expressed in two major indexes: richness and evenness. Richness refers to the number of different species inhabiting a given area, and evenness refers to the similarity in the number of species inhabiting a given area. The α-diversity of each sample was estimated using Shannon’s diversity index computed considering both the richness and evenness [14] using the diversity core-metrics-phylogenetic plugin in QIIME2. β-diversity refers to the diversity by measuring the species change between two given areas. Multidimensional scaling (MDS), representing the compositional dissimilarity between samples or groups, was computed using the Bray–Curtis dissimilarity matrix, and the MDS plot was visualized in R studio.

### 2.6. Bacterial Composition and Relative Abundance Analysis

To observe the bacterial composition of the gut microbiome, bacterial composition bar plots were visualized using R studio. To identify markers that differed in abundance between the groups, LDA effect size (LEfSe) analysis was conducted using the microbiomeMarker package in R studio. The LEfSe analysis was set as normalization = counts per million mapped reads (CPM), Kruskal–Wallis test cut-off = 0.01, Wilcoxon test cut-off = 0.01, LDA (linear discrimination analysis (LDA) score cut-off = 4. Subsequently, the abundance plot, LDA score bar chart, and cladogram were visualized based on the LEfSe analysis results in R Studio. Additionally, based on the significant markers extracted from the LEfSe analysis, changes in the abundance of each marker according to probiotic administration were comparatively analyzed with a linear mixed model (LMM) using the lme4 package in R Studio.

## 3. Results

### 3.1. Growth Performance

**Figure 1:**
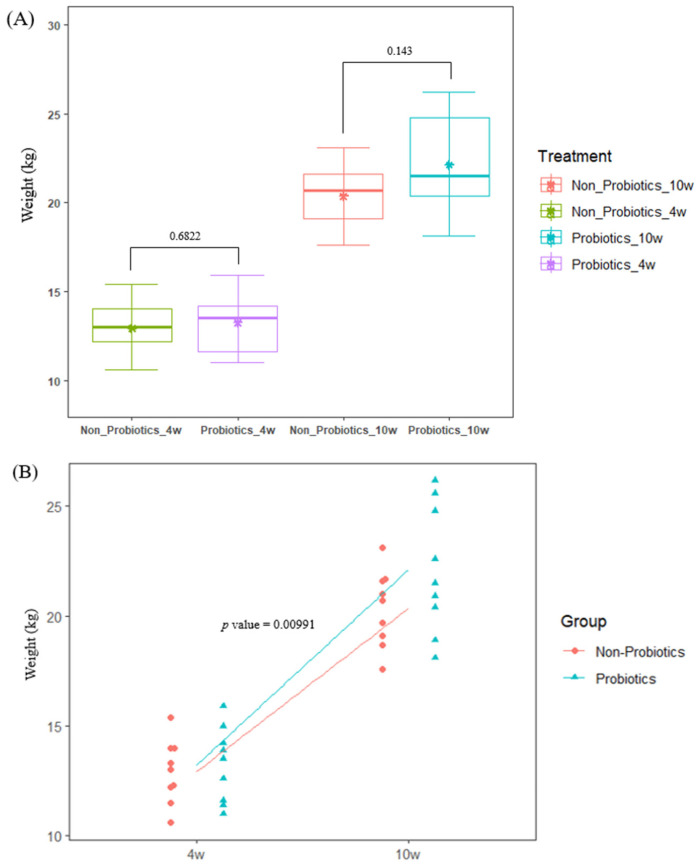
Growth performance of piglets according to probiotics supplementation. (**A**) Box plot of mean body weight by group. There were no significant differences between groups (*p* > 0.05). * indicates mean of each group. (**B**) Linear mixed-model analysis for growth performance. The PrB tended to significantly increase body weight gain compared to the N-PrB (*p* = 0.00991).

**Figure 2:**
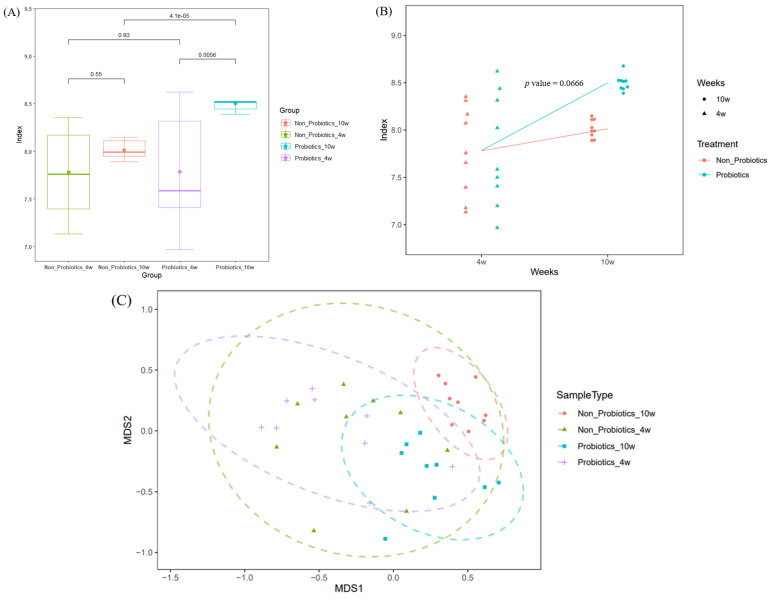
Microbial diversity analysis according to probiotics supplementation. (**A**) The box plot of Shannon’s diversities by group. The PrB exhibited the significantly higher Shannon’s diversity indexes compared to the N-PrB. * indicates mean of each group. (**B**) Linear mixed-model analysis for Shannon’s diversity changes according to the probiotic supplementation. The PrB exhibited a significant increasing tendency of Shannon’s diversity compared to the N-PrB at the significance level of *p* < 0.01. (**C**) The MDS plot for β-diversity analysis computed by Bray–Curtis dissimilarity matrix. The 95% confidence interval ranges (dotted lines) of both groups were more clearly separated at 10 weeks compared to 4 weeks.

No clinical symptoms were observed in piglets during the experimental period. When comparing the mean BWs of each group using the *t*-test, there was no significant difference between the groups at 4 weeks. PrB showed an increase in mean BW compared to N-PrB at 10 weeks; however, there was no significant difference between the groups (Table 1 and Figure 1A). However, there was a significant difference between the groups in the linear mixed-model analysis (LMM) of the weight gain tendency by group during the experimental period (Figure 2A). The BWs of PrB tended to increase significantly compared to that of N-PrB (*p* = 0.00991). From these results, we determined that probiotic supplementation was beneficial for the growth performance of piglets.

### 3.2. Microbial Diversity

In the α-diversity analysis with Shannon’s diversity, the 4-week-old piglets showed somewhat large variation in the indexes, but the deviations of the indexes within the groups decreased in the 10-week-old piglets. Indices by group were statistically analyzed using the Wilcoxon rank-sum test. There were no significant differences between the groups at 4 weeks, but there was a significant difference between the groups at 10 weeks (Figure 2A). PrB exhibited significantly higher indices than N-PrB (*p* = 4.1 × 10^−5^). In addition, in the LMM analysis of Shannon’s diversity index, the indices of PrB tended to increase significantly compared to those of N-PrB (*p* < 0.01; *p* = 0.0666) (Figure 2B). Thus, it is determined that probiotics supplementation was beneficial to the α-diversity of piglets.

In the β-diversity analysis with MDS, at 4 weeks, 95% confidence interval ranges of each group were broad, and most parts overlapped each other. However, at 10 weeks, these ranges narrowed and became separated from each other after 4 weeks (Figure 2C).

### 3.3. Bacterial Composition

**Figure 3:**
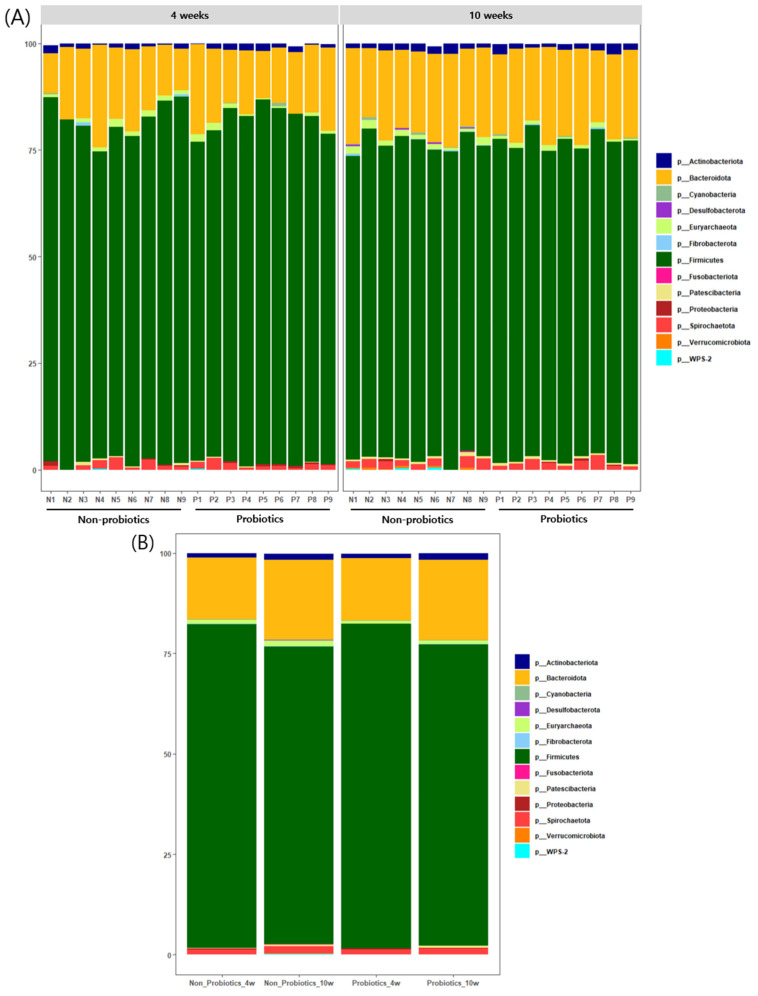
The bar charts of bacterial composition in phylum level by individual (**A**) or group (**B**). The most abundant bacteria were *p_Fimicutes*, followed by *p_Bateroidota* and these bacteria accounted for most of the fecal microbiome of the piglets. When comparing them at 4 and 10 weeks, *p_Fimicutes* slightly decreased, on the other way *p_Bateroidota* slightly increased in both groups as time passed.

Several bacteria were observed at the phylum level in the bar charts of the bacterial composition. Among these bacteria, the most abundant were *p_Firmicutes*, followed by *p_Bacteroidota*, which accounted for most of the fecal microbiome of the piglets throughout the experimental period. When comparing weeks 4 and 10, *p_Firmicutes* slightly decreased, whereas *p_Bacteroidota* slightly increased in both groups over time (Figure 3).

**Figure 4:**
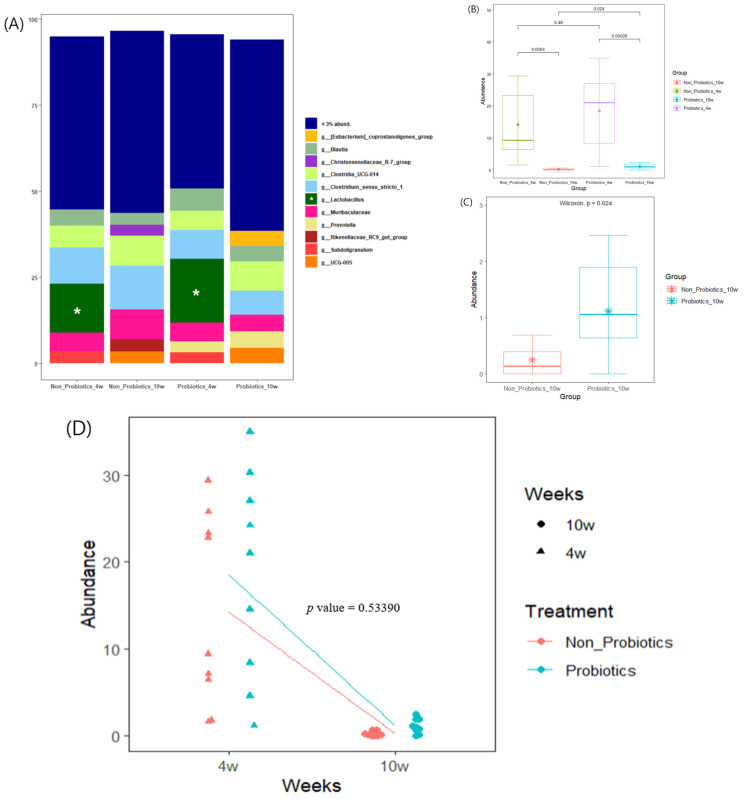
Bacterial composition analysis in genus level. In genus level, severe reductions of *lactobacillus* were observed in both group when comparing at 4 and 10 weeks (**A**). There was no significant difference between groups at 4 weeks (**B**,**C**), but a significant difference between groups at 10 weeks (**C**). No significance was observed in the LMM analysis on the *lactobacillus* abundance by probiotics supplementation and time (*p* = 0.53390) (**D**).

At the genus level, severe reductions in *Lactobacillus* were observed in both groups as time passed from 4 to 10 weeks. The mean *Lactobacillus* abundances of PrB and N-PrB accounted for a relatively high proportion (PrB: 18.5%, N-PrB: 14.2%) at 4 weeks (Figure 4A), whereas the abundances in both groups were severely reduced below 3%. Although there was a significant difference between the two groups according to the Wilcoxon rank-sum test at 10 weeks (*p* = 0.024) (Figure 4B,C), no significant difference was observed in the LMM analysis of *Lactobacillus* abundance according to probiotic supplementation and time (*p* = 0.53390) (Figure 4D). Thus, supplementation with the probiotic combination did not alleviate the *Lactobacillus* reduction in this study.

### 3.4. LEfSe Analysis to Find Significant Markers

**Figure 5:**
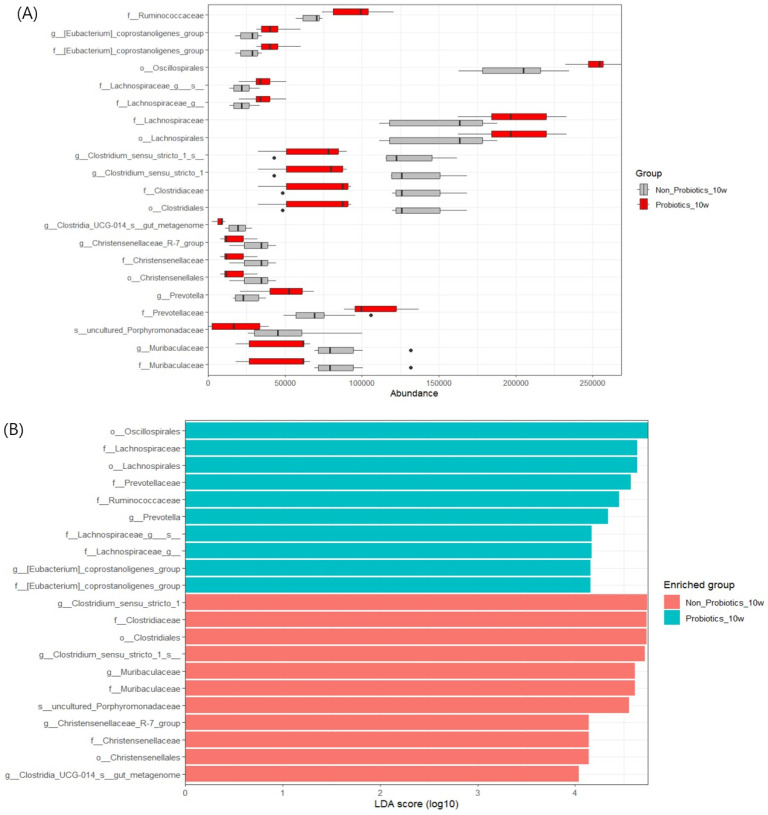

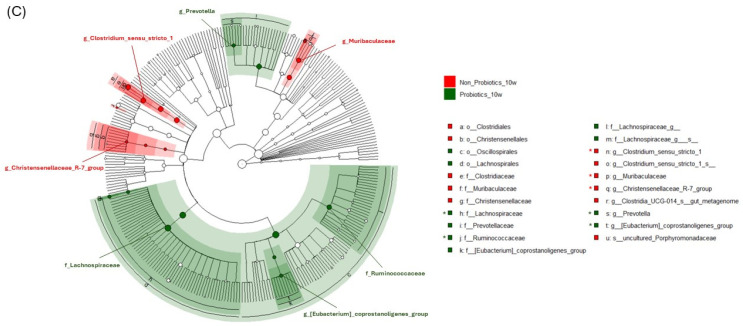
LDA effect size (LEfSe) analysis. The abundance plot on count reads normalized by CPM (**A**), LDA score bar chart (**B**) and cladogram (**C**) were visualized based on the significant markers (bacteria differing in abundance between the groups) at 10 weeks. * indicates the representative bacteria with the highest taxonomic rank on a darker background color, indicating higher abundance in each group (red: N-PrB, green: PrB). The *f_Lachnospiraceae*, *f-[Eubaterium]_coprostanoligenes_group* and *f_Ruminococcaceae* lineage were significantly abundant in the PrB; on the other hand, the *o_Christensenellales*, *o_Clostridiales* and *f_Muribaculaceae* lineage were significantly abundant in the N-PrB.

In the LEfSe analysis, no markers (bacteria with different abundances between the two groups) were observed at 4 weeks, whereas several markers were found at 10 weeks. The *f_Lachnospiraceae*, *f_Eubacterium coprostanoligenes_group* and *f_Ruminococcaceae* lineages were significantly abundant in PrB, whereas the *o_Christensenellales*, *o_Clostridiales*, and *f_Muribaculaceae* lineages were significantly abundant in N-PrB (Figure 5).

### 3.5. LMM Analysis on Relative Abundance Changes

**Figure 6:**
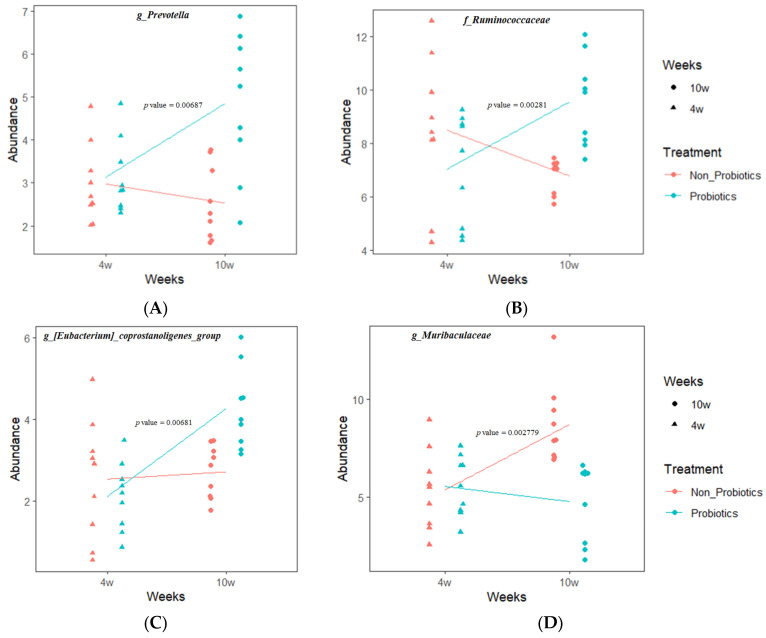
LMM analysis on relative abundance changes according to age and probiotics supplementation. *g_Prevotella* (*p* = 0.00687) (**A**), *f_Ruminococcaceae* (*p* = 0.00281) (**B**) and *g_Eubacterium_coprostanoligenes_group* (*p* = 0.00681) (**C**) tended to significantly increase in the PrB compared to the N-PrB, whereas *g_Muribaculaceae* (*p* = 0.002779) (**D**) tended to significantly increase in the N-PrB compared to the PrB.

LMM analysis was performed on the relative abundance changes of the significant markers identified by LEfSe analysis according to age and probiotic supplementation. Based on the cladogram, significant markers with the highest taxonomic rank in a darker background color, indicating higher abundance, were selected. Finally, *f_Lachnospiraceae*, *g_Eubacterium coprostanoligenes_group*, *f_Ruminococcaceae*, *g_Prevotella* (higher abundance in PrB), *g_Christensenellaceae R-7 group*, *g_Clostridium sensu stricto 1*, and *g_Muribaculaceae* (higher abundance in N-PrB) were analyzed. As a result, *f_Ruminococcaceae* (*p* = 0.00281) and *g_Prevotella* (*p* = 0.00687) tended to increase significantly in PrB but decreased in N-PrB. *g_Eubacterium coprostanoligenes group* exhibited an increasing tendency in both groups, but it tended to increase significantly in the PrB group compared with the N-PrB group (*p* = 0.00681). *g_Muribaculaceae* tended to significantly increase in N-PrB, but decreased in PrB (*p* = 0.002779) (Figure 6). No significant differences were found in the other selected significant markers (Supplementary Figure S1).

## 4. Discussion

Probiotics are live, non-pathogenic microorganisms that improve microbial balance, particularly in the gastrointestinal tract, and have been widely used to provide benefits for animal health and ultimately improve productivity [1,2]. Several bacterial and yeast genera, including *Enterococcus*, *Bacillus*, *Lactobacillus*, *Bifidobacterium*, and *Saccharomyces* have been investigated and used as probiotics [3,15,16,17].

Human-origin probiotics have been widely used for animal use, but different host-origin probiotics may have reduced effectiveness due to different gut environment. Intestinal colonization and physiological activities of microorganisms will be most optimized when in their natural habitat. Thus, host-derived microorganisms can be the most appropriate probiotic source [18,19]. For that reason, two pig-origin probiotic bacteria strains (*E. faecium* EF-1 and *B. subtilis* BS-CTC), which were originated from swine feces, was used for the study.

In many previous studies, these two probiotic species was proven to be safe and effective on improving growth performance [20,21,22,23]. *S. cerevisiae* is one of the probiotic yeasts. Its safety and effectiveness to improve growth performance have been well investigated like the above two bacteria [24,25]. In fact, the above three probiotics have been studied as feed additives for a long time and large-scale fermentation for these probiotics is well established in most production companies. Thus, it is expected to be advantageous in terms of development and production costs when commercialized.

Currently, in most research or commercial products, various formulations by combining different strains have been utilized for complementary activities that may lead synergistic advantages [26]. The effects of single use of the above three probiotics have been well investigated [20,21,22,23,24,25], but no studies could be found on the application of the three probiotics combination to piglets. Thus, in this study, the three probiotics combination was prepared and applied to the weaned piglets.

The nursery stage is an important period in pig production systems. Animals never get over a bad start; thus, it is critical to provide weaned piglets with optimal control. However, weaned piglets experience many stresses, such as feed changes due to weaning events and growing environment changes, which can lead to diarrhea, reduced growth rate, changes in gut morphology, and increased susceptibility to disease and death [7,8]. This stressful circumstance may induce an imbalance in the gut microbiota of weaned piglets. In fact, 4-week-old piglets exhibited a wide range of Shannon diversity indices, reflecting the unstable status of the gut microbiome in early weaned piglets. However, these deviations in α-diversity were decreased as age increased from 4 to 10 weeks and it appears to have stabilized naturally. This stabilization process was observed in the β-diversity analysis. The wide range of the 95% CI for each group also narrowed as age increased from 4 to 10 weeks in the MDS plot. In the LMM analysis on α-diversity, both piglets with and without probiotics exhibited a tendency to increase microbial diversity as age increased from 4 to 10 weeks, but the microbial diversity of the piglets with probiotics tended to increase significantly compared to the piglets without probiotics (*p* < 0.1). Generally, abundant gut microbial diversity reflects a healthy status [27]; thus, probiotic supplementation had a positive impact on the establishment of healthy gut microbiota, which appeared to have led to an improvement in growth performance.

The structure of the gut microbiota can be affected by the environment and diet, resulting in individual deviations. However, many previous studies have revealed that *Firmicutes* and *Bacteroidota* are the major core bacteria in the gut microbiota at the phylum level, regardless of age [28,29]. In this study, the most abundant phylum was *Firmicutes*, followed by *Bacteroidota*, which accounted for over 90% of the gut microbiota of the piglets, regardless of the group or individual, throughout the entire experimental period, and the structures of gut microbiota at the phylum level were very similar to each other. Other phyla occupied a small part, and *Proteobacteria*, *Spirochaetes*, and *Campilobacterota*, which are known markers of microbial dysbiosis, gut inflammation, and disease, were rarely found in the gut microbiota. These consistent compositions imply that all piglets tested in this study remained healthy without severe disease during the experimental period [30].

Interesting observations were made at the genus level. A severe reduction in *Lactobacillus* was observed as age increased from 4 to 10 weeks in all piglets, regardless of probiotic supplementation. This result was consistent with that of Chen et al., who reported that *Lactobacillus* decreased from 12.25% (10 d before weaning) to 1.76% (21 d after weaning) [28]. *Lactobacillus*, which has nutrient uptake abilities from milk oligosaccharides and host-derived glycans, is one of the predominant genera in the gut microbiota of suckling piglets and gradually decreases in abundance during the nursery stage after weaning [28,31,32]. Facultative anaerobes, such as *Lactobacilli* and *E. coli* colonize the gut immediately after birth and gradually grow to high abundances, resulting in an oxygen-deprived environment during weaning. This anaerobic environment leads to the expansion of obligate anaerobes, which are typically specialized to degrade plant-derived complex polysaccharides [32,33]. It was revealed that *Ruminococcus* and *Prevotella* spp., which are groups of obligate anaerobic bacteria degrading fiber [34,35], show higher abundance after weaning [28,36,37]. In the present study, the relative abundance of *Lactobacillus* was significantly higher in 10-week-old piglets provided probiotics than in 10-week-old piglets without probiotics. However, it was determined by LMM analysis that the probiotic formulation used in this study failed to alleviate the severe decline, which seems to be attributed to the severe reduction in *Lactobacillus* observed in both groups. The beneficial effects of *Lactobacillus* on swine production are well known [38]; therefore, conserving *Lactobacillus* abundance after weaning may help improve productivity. Further studies are required to resolve these issues.

The gut microbiota is essential for degrading fiber by fermentation, producing metabolites due to the lack of digestive enzymes for fibers in pigs [39]. Weaned piglets continue to mature their gut microbiota, favoring feed containing abundant fiber, which is advantageous for growth in the process of feed adaptation and faster maturation [32]. In this study, *Ruminococcaceae* and *Prevotella* significantly increased in piglets treated with probiotics compared to those not treated with probiotics during the experimental period (*p* < 0.05). *Ruminococcaceae* and *Prevotella* can ferment plant-derived non-starch polysaccharides to SCFA [40,41] and are commonly found in high abundance after weaning [28,36]. Moreover, previous studies have revealed that these bacteria are significantly more abundant in healthy piglets with a high average daily gain or without post-weaning diarrhea [30,32,36]. Thus, it seems that piglets supplemented with probiotics sustained healthier gut microbiota, leading to higher digestive capability and nutrient intake, compared to piglets without probiotics.

In this study, *Eubacterium coprostanoligenes* was also significantly increased in piglets with probiotics compared to those without probiotics (*p* < 0.05). *E. coprostanoligenes* biotransforms cholesterol to coprostanol, which is mainly excreted via feces and is therefore related to the cholesterol elimination process by microorganisms in the body. Cholesterol is the main component of cell membrane architecture and steroid hormones in animals. However, excessive serum cholesterol is likely to have a negative effect on health [42]. Previous studies have reported that piglets with lower serum cholesterol levels show enhanced growth performance and immune responses compared to piglets with higher serum cholesterol levels [43,44]. Thus, an increase in *Eubacterium coprostanoligenes* may help control serum cholesterol levels in pigs. However, *Muribaculaceae* significantly increased in piglets without probiotics compared to piglets with probiotics (*p* < 0.05) in this study. *Muribaculaceae*, belonging to the phylum *Bacteroidota*, was named in 2019, and its function has not been well investigated because of difficulties in cultivation [45]. Further research is required to determine the roles of these bacteria in pigs.

## 5. Conclusions

In the present study, weaned piglets were fed a probiotic combination of *E. faecium*, *B. subtilis*, and *S*. *cerevisiae* during the nursery stage (6 weeks). The long-term supplementation with probiotics had beneficial effects on the gut microbiota of weaned piglets. In the piglets with probiotics, the α-diversity of the gut microbiota significantly increased. Additionally, the abundances of *Ruminococcaceae*, *Prevotella*, and *E. coprostanoligenes* were significantly increased, but not in the piglets without probiotics. These beneficial changes in gut microbiota ultimately led to improved growth performance. Thus, the long-term supplementation with probiotic combination is highly recommended for piglets in the nursery stage after weaning.

## Figures and Tables

**Table 1:** Body weights and weight gains of piglets during the experimental period.

Probiotics				Non-Probiotics			
Animal ID	Body Weight (kg)		Weight Gain (kg)	Animal ID	Body Weight (kg)		Weight Gain (kg)
	4 w	10 w			4 w	10 w	
74-7	11.0	18.1	7.1	77-61	14.0	20.7	6.7
73-45	11.4	18.9	7.5	74-61	12.3	19.1	6.8
73-7	12.6	20.4	7.8	73-04	10.6	17.6	7.0
73-12	13.5	21.5	8.0	72-86	11.5	18.7	7.2
72-80	13.9	22.6	8.7	75-37	12.2	19.7	7.5
75-82	11.6	20.9	9.3	74-41	14.0	21.7	7.7
73-16	15.9	26.2	10.3	74-32	15.4	23.1	7.7
73-72	15.0	25.6	10.6	73-75	13.0	21.0	8.0
71-4	14.2	24.8	10.6	74-51	13.3	21.6	8.3
Average	13.2	22.1	8.9	Average	12.9	20.4	7.4
SEM	0.6	1.0	0.5	SEM	0.5	0.6	0.2

## Data Availability

All data generated or analyzed during this study are included in this published article and its additional files. The datasets analyzed in the current study are available from the corresponding author upon reasonable request.

## Supplementary Materials

The following supporting information can be downloaded at: https://www.mdpi.com/article/10.3390/ani14111652/s1, Figure S1: LMM analysis on relative abundance changes according to age and the probiotics supplementation.
