# Supplementary material for: Impact of Long-Term Supplementation with Probiotics on Gut Microbiota and Growth Performance in Post-Weaned Piglets
Source: Animals (Basel). 2024 May 31;14(11):1652. doi: 10.3390/ani14111652 (PMC11171352; doi:10.3390/ani14111652)
Supplement: Supplementary file 1 [file animals-14-01652-s001.zip › animals-3024131-supplementary.pdf]

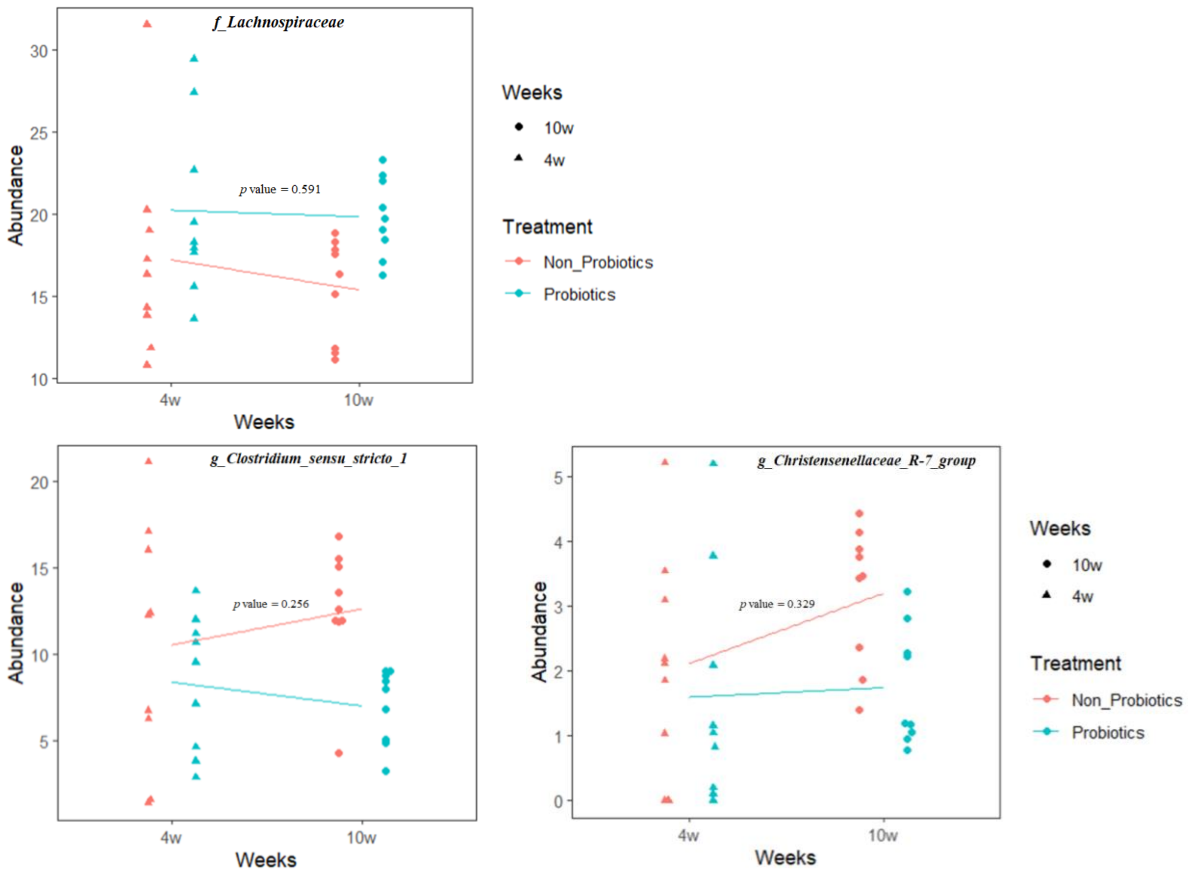

Supplementary Figure S1. LMM analysis on relative abundance changes according to age and the probiotics supplementation. *f_Lachnospiraceae* (A), *g_Clostridium_sensu_ stricto_1* (B) and *g_Christensenellaceae_R-7_group* (C) did not show significant differences between the groups.
